# USP40 promotes hepatocellular carcinoma cell proliferation, migration and stemness by deubiquitinating and stabilizing Claudin1

**DOI:** 10.1186/s13062-024-00456-3

**Published:** 2024-02-02

**Authors:** Qingsong Wu, Yuanyuan Qiu, Jinhui Guo, Zibo Yuan, Yingnan Yang, Qingwei Zhu, Zhe Zhang, Junwei Guo, Yanfang Wu, Junyu Zhang, Dongsheng Huang, Kangsheng Tu, Xiaoge Hu

**Affiliations:** 1 The Second Clinical Medical College of Zhejiang Chinese Medical University, Hangzhou, 310053 China; 2 The Key Laboratory of Tumor Molecular Diagnosis and Individualized Medicine of Zhejiang Province, Zhejiang Provincial People’s Hospital (Affiliated People’s Hospital), Hangzhou Medical College, Hangzhou, 310014 China; 3 Department of Oncology, Teng Zhou Central People’s Hospital Affiliated to Jining Medical College, Tengzhou, 277500 China; 4 The Medical College of Qingdao University, Qingdao, 266000 China; 5 Department of Hematology, The First People’s Hospital of Fuyang Hangzhou, Hangzhou, 311402 China; 6 Department of Hematology, Lishui Central Hospital of Zhejiang Province, Lishui, 323020 China; 7 Department of Hepatobiliary Surgery, The First Affiliated Hospital of Xi’an Jiaotong University, Xi’an, 710061 China; 8 General Surgery, Cancer Center, Department of Hepatobiliary and Pancreatic Surgery and Minimally Invasive Surgery, Zhejiang Provincial People’s Hospital (Affiliated People’s Hospital), Hangzhou Medical College, Hangzhou, 310014 China

**Keywords:** USP40, Claudin1, HCC, Deubiquitinating

## Abstract

**Background:**

Hepatocellular carcinoma (HCC) is a prevalent malignant tumor that poses a major threat to people’s lives and health. Previous studies have found that multiple deubiquitinating enzymes are involved in the pathogenesis of HCC. The purpose of this work was to elucidate the function and mechanism of the deubiquitinating enzyme USP40 in HCC progression.

**Methods:**

The expression of USP40 in human HCC tissues and HCC cell lines was investigated using RT-qPCR, western blotting and immunohistochemistry (IHC). Both in vitro and in vivo experiments were conducted to determine the crucial role of USP40 in HCC progression. The interaction between USP40 and Claudin1 was identified by immunofluorescence, co-immunoprecipitation and ubiquitination assays.

**Results:**

We discovered that USP40 is elevated in HCC tissues and predicts poor prognosis in HCC patients. USP40 knockdown inhibits HCC cell proliferation, migration and stemness, whereas USP40 overexpression shows the opposite impact. Furthermore, we confirmed that Claudin1 is a downstream gene of USP40. Mechanistically, USP40 interacts with Claudin1 and inhibits its polyubiquitination to stabilize Claudin1 protein.

**Conclusions:**

Our study reveals that USP40 enhances HCC malignant development by deubiquitinating and stabilizing Claudin1, suggesting that targeting USP40 may be a novel approach for HCC therapy.

**Supplementary Information:**

The online version contains supplementary material available at 10.1186/s13062-024-00456-3.

## Introduction

Hepatocellular carcinoma (HCC) is a worldwide prevalent malignant tumor of the digestive system, with the sixth incidence and the third mortality rate of malignant tumors [1]. Generally speaking, the prognosis for HCC is extremely dismal, with a 5-year survival rate of 15–19% in North American countries, but just 12.1% in China [2, 3]. The treatment of HCC includes surgical resection, local ablation, liver transplantation, transhepatic artery interventional therapy, targeted therapy, and immunotherapy [4]. Due to the absence of specific symptoms in the initial phase of HCC, the majority of patients have reached the middle and late stages when diagnosed, mainly relying on intervention, targeted, and immunotherapy.

Previous studies have found that multiple deubiquitinating enzymes (DUBs) are associated with the progression of HCC. Ubiquitin-specific protease 40 (USP40) is a novel DUB belonging to the USP family. Studies have shown that the single nucleotide polymorphism of USP40 is associated with Parkinson’s disease and inflammatory bowel disease [5–7]. Takagi et al. reported that knockdown of USP40 interfered with glomerulogenesis in zebrafish [8]. In addition, USP40 was involved in the process of glomerulosclerosis by deubiquitinating HINT1 and stabilizing p53 leading to podocyte hypertrophy [9]. In neoplastic diseases, USP40 mutations are associated with clinicopathological features such as poor survival in patients diagnosed with clear cell renal cell carcinoma (ccRCC), and can be used as diagnostic, prognostic and therapeutic markers for ccRCC [10]. In non-small cell lung cancer (NSCLC), USP40 suppresses polyubiquitination of anti-apoptotic protein CFLAR_L_, and GMEB1 promotes CFLAR_L_ stability via USP40, followed by inhibition of CASP8 precursor activation and apoptosis [11]. However, the function of USP40 in HCC remains to be elucidated.

Claudin1, a tight junction protein family member, is mainly located in both tight junctions and non-junctions such as the cytoplasm and nucleus. Studies have shown that Claudin1 is able to mediate HCV virus infection into hepatocytes [12]. In addition, Claudin1 has been reported to be overexpressed in various kinds of tumor tissues and is directly involved in the development and progression of many malignancies, such as colon cancer, head and neck squamous cell carcinoma, breast cancer and HCC [13–17]. In HCC, Claudin1 was demonstrated to be related to the epithelial-mesenchymal transition (EMT), cell migration and invasion [18, 19].

In this work, we observed that USP40 was elevated in HCC tissues compared to normal tissues. USP40 overexpression dramatically accelerated HCC cell growth, migration and stemness both in vitro and in vivo. Moreover, Claudin1 was regarded as a target of USP40. As a DUB, USP40 could interact with and stabilize Claudin1 in HCC. Functional rescue results further demonstrated that USP40 and Claudin1 formed an oncogenic axis to accelerate HCC development. Our study indicates that USP40 could serve as a novel therapeutic target for HCC.

## Materials and methods

### Cell culture

The human normal liver cell line LO2 and HCC cell lines Huh7, MHCC97H, Hep3B, SNU387 and HepG2 cells were supplied by the Cell Bank of the Chinese Academy of Sciences. Hep3B and SNU387 cells were grown in MEM medium and RPMI 1640 medium, respectively. All other cell lines mentioned above were grown in high glucose DMEM. The media were all added with 10% fetal bovine serum and 1% penicillin/streptomycin. All cells were cultured in a 37 °C incubator with 5% CO_2_.

### Antibodies and reagents

The antibodies listed below were utilized: USP40 (cat# sc-514248, Santa Cruz), USP40 (ab121234, Abcam), Claudin1 (cat# 13255, Cell Signaling Technology), Claudin1 (cat# sc-166338, Santa Cruz), Claudin1 (cat# 13050-1-AP, Proteintech), HA-tag (cat# 51064-2-AP, Proteintech), c-Myc (ab32072, Abcam), KLF4 (cat# 12173, Cell Signaling Technology), Ub (cat# sc-8017, Santa Cruz), and β-actin (cat# 66009-1-Ig, Proteintech). Cycloheximide (CHX, cat# S7418) and MG132 (cat# S2619) were purchased from Selleck.

### Lentivirus infection and cell transfection

For infection, lentiviruses with short hairpin RNA (shRNA) and USP40 overexpression sequences were constructed by GenePharma (Shanghai, China). The cells were planted in a six-well dish and cultivated overnight. After rinsing with PBS, 1 mL of fresh medium was added, followed by the addition of the lentivirus to the medium (MOI = 20). The medium volume was increased to 2 mL the next day. 48 h after infection, the medium with lentivirus was aspirated and substituted with new medium. After 72 h, the culture medium was supplemented with puromycin to obtain stable infected cell lines by screening for 1 week. Finally, total protein was collected from stably infected cell lines to evaluate knockdown and overexpression efficiency. The shRNA sequences utilized in the study were presented in Additional file 1: Table S1.

For transfection, scramble small interfering RNA (siRNA), Claudin1 siRNA and plasmid were bought from GenePharma. According to the instructions of transfection reagent, 10 μL (20 μM) siRNA or 2 µg plasmid were transfected into cells by applying lipofectamine 3000. The siRNA sequences used for transfection were listed in Additional file 1: Table S2.

### Cell counting kit-8 (CCK-8)

In 96-well plates, 3 × 10^3^ Huh7 and MHCC97H cells were cultivated to determine cell proliferation. Following 24, 48, and 72 h of growth, 10 µL CCK-8 detection solution (cat# 40203ES80, Yeasen Biotechnology) was added to each well, and the absorbance was recorded at 450 nm after 2 h of incubation.

### Colony formation

For colony formation assays, 500 Huh7 and MHCC97H cells were planted in 6-well dishes and cultivated for 8 days. The formed colonies were first fixed for 30 min with 4% paraformaldehyde (G1101, Servicebio), followed by 30 min of dyeing with 0.5% crystal violet (MB4721, Meilunbio), and lastly colonies (> 50 cells) were counted by microscope.

### Wound healing

In 6-well plates, cells were planted and cultured until they were fully confluent. The confluent monolayer was scraped with a clean pipette tip and rinsed three times with PBS to eliminate free cells. At regular time intervals, the area of the cells migrated across the wound was measured with Invitrogen EVOS M7000 microscope. Images were analyzed using Image J software.

### Transwell assay

200 µL of serum-free medium with 2 × 10^4^ cells was seeded to the top chamber of the transwell and 700 µL of complete medium was placed to the bottom chamber. After 24 h of growth, the migrated cells were fixed with 4% paraformaldehyde. Then they were dyed with crystal violet for 30 min and finally photographed with Invitrogen EVOS M7000 microscope.

### Sphere formation

5 × 10^4^ HCC cells were placed in ultra-low binding 6-well dishes and grown in DMEM/F12 medium. Animal-Free Recombinant Human EGF (AF-100-15, Peprotech), Recombinant Human FGF-basic (100-18B, Peprotech) and N-2 supplement (17502-048, ThermoFisher) were added to the medium to ensure cell sphere formation. When the spheres formed by the cells grew to 8 days, they were viewed and photographed using a microscope.

### Real-time quantitative PCR (RT-qPCR)

Following the producer’s directions, total RNA was isolated from cells by utilizing the RNA-Quick Purification Kit (cat# RN001, ES science). NanoDrop One (ThermoFisher Scientific) was applied to detect the concentration and purity of RNA. Subsequently, using RNA as a template, reverse transcription was conducted using PrimeScript™ RT Reagent Kit (cat# RR037A-1, Takara) to synthesize complementary DNA (cDNA). Real-time PCR was carried out with SYBR Green (cat# 11184ES08, Yeasen Biotechnology) following the manufacturer’s directions through a 7500 real-time PCR device under the following procedures: 95 °C for 2 min, 95 °C for 10 s, 60 °C for 30 s, The melting curve stage is 95 °C for 15 s, 60 °C for 30 s, and 95 °C for 5 s, a total of 40 cycles. Each experiment was conducted three times. The relative RNA expression of USP40 and Claudin1 was calculated by 2^−ΔΔCt^ method. The primer sequences used for RT-qPCR were shown in Additional file 1: Table S3.

### Western blotting

Protein lysates incorporating PMSF and protease inhibitors were utilized to collect total protein from cells and tissues. Protein quantification was performed using a PierceBCA protein assay kit to determine protein concentration. After boiling at 95 °C for 10 min, equal quantities of proteins (20–30 µg) were isolated by 10% SDS-PAGE gel electrophoresis and transferred to PVDF membranes. The PVDF membranes were then blocked with 5% skimmed milk for 1 h at room temperature. After washing, the membranes were divided into small pieces based on the molecular weight of the target protein using a pre-stained page ruler. Then the membranes were incubated with the corresponding primary antibodys overnight at 4 °C. Following three washes with TBST, the membranes were incubated with HRP-conjugated secondary antibodies for 1 h at room temperature. Finally, the membranes were washed three times with TBST and visualized with ECL reagent (Bio-Rad).

### CHX, immunoprecipitation (IP), and ubiquitination assays

The half-life of Claudin1 was examined using the CHX assay. Briefly, protein synthesis inhibitor CHX (100 µg/mL) was administered to the cells for different times. After being treated with CHX for 0, 2, 4, or 6 h, the cells were harvested. Next, proteins were collected from the treated cells and western blotting was applied to test Claudin1 expression.

For IP experiments, HA-USP40 and Claudin1 plasmids were co-transfected into Huh7 and MHCC97H cells for 48 h. Magnetic beads were added to the indicated antibody working solution and incubated at room temperature for 15 min. Total protein was collected from HCC cells using IP lysis buffer, then the magnetic bead-antibody complex was added to the cell lysate and incubated at 4 °C overnight. The next day, the supernatant was removed, and the remaining magnetic bead-antigen–antibody complexes were preserved and resuspended with 1 × loading buffer, then heated at 95 °C for 10 min. Finally, the beads were removed and the residual immunoprecipitated protein complexes were identified by western blotting.

To assess Claudin1 ubiquitination, proteasome inhibitor MG132 (10 µM) was administered to the cells for 8 h, then proteins were collected and assayed by IP.

### Immunofluorescence staining

5 × 10^4^ MHCC97H cells were placed in glass-bottomed cell culture dishes for 24 h. The cells were fixed with 4% paraformaldehyde for 30 min, permeated with 0.5% Triton-X for 10 min, and then blocked with goat serum for 1 h at room temperature. Primary antibodys were added to the cells overnight at 4 °C. Following three washes with cold PBS, the cells were then incubated with fluorescent conjugated secondary antibodys for 1 h. Finally, Antifade Mounting Medium with DAPI (P0131, Beyotime) was added and observed under a confocal microscope (Leica TCS SP8).

### In vivo tumorigenesis assay

Twenty female BALB/c nude mice (3–4 weeks old) were bought from Hangzhou Hangsi Biotechnology and maintained in a specific pathogen-free (SPF) animal center. Mice were allocated into four groups at random (n = 5 per group). 1.5 × 10^7^ MHCC97H cells stably expressing shUSP40, shNC, USP40 or NC were subcutaneously injected into each mouse. Tumor growth was recorded every 5 days. The following is the tumor volume (V) calculation formula: tumor volume (mm^3^) = (length × width^2^)/2. The mice were euthanized after injection for 30 days, and tumors were isolated and weighed.

### HCC tissues and immunohistochemistry (IHC)

Twenty pairs of HCC and corresponding adjacent tissues were obtained from the Department of Pathology of Zhejiang Provincial People’s Hospital. IHC procedures were carried out as previously reported [20]. Two experienced pathologists scored the staining intensity and percentage of positive cells in a double-blind manner. The scoring criteria were based on previously published literature [21].

### Statistical analysis

GraphPad Prism 9.0 was adopted for statistical analysis. The data are expressed as mean ± SEM. Student’s t-test was performed to compare two groups of independent samples. One-way ANOVA was applied to compare three or more groups. Each experiment was carried out at least three times. *P* < 0.05 was regarded as statistically significant.

## Results

### USP40 is elevated in HCC and predicts poor prognosis

**Fig. 1:**
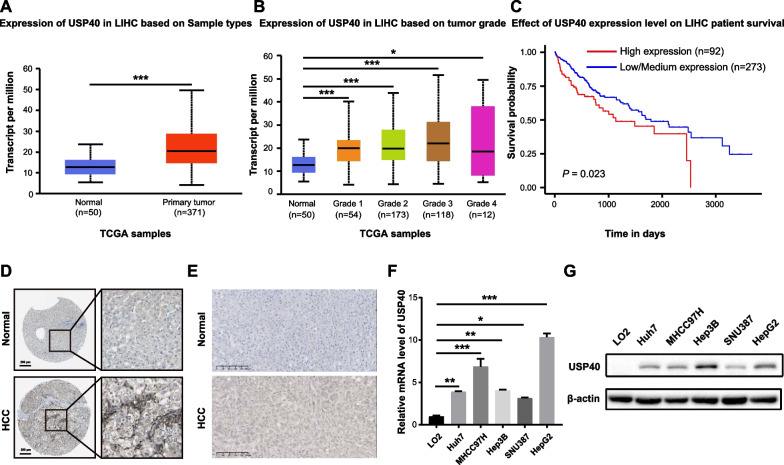
USP40 is elevated in HCC and predicts poor prognosis. **A**–**B** Compared to liver normal tissues, USP40 expression was drastically elevated in HCC tissues and correlated with tumor stage. **C** Kaplan–Meier plotter analysis showed that increased USP40 expression was associated with worse survival rate in HCC patients. Data were generated from the TCGA and UALCAN databases. **D** IHC images of USP40 expression in liver normal tissues and HCC samples were gained from the Human Protein Atlas (HPA) database. Scale bar, 200 μm. **E** Representative IHC staining pictures of USP40 were displayed in HCC specimens and normal tissues. Scale bar, 200 μm. **F** RT-qPCR and **G** western blotting results showed that USP40 was overexpressed in HCC cell lines (Huh7, MHCC97H, Hep3B, SNU387, and HepG2) compared to LO2 in mRNA and protein levels. LIHC: liver hepatocellular carcinoma. Data were displayed with typical pictures of three independent experiments. *P < 0.05, **P < 0.01, ***P < 0.001

Through TCGA and UALCAN databases, we discovered that USP40 mRNA was elevated in tumor compared to normal tissues and correlated with tumor stage (Fig. 1A, B). In addition, using UALCAN database and Kaplan–Meier survival analysis, we found that USP40 overexpression predicted poor prognosis in HCC patients (Fig. 1C). Furthermore, by analyzing the IHC data in the HPA database, we observed that USP40 protein was overexpressed in HCC samples compared to normal tissues (Fig. 1D). Consistent with the conclusion in the HPA database, our IHC outcomes verified that USP40 expression was higher in HCC specimens than in normal tissues (Fig. 1E). Then, we examined USP40 expression in HCC cell lines. The findings displayed that USP40 expression was upregulated in HCC cell lines compared to that in liver normal cell line LO2 at both mRNA and protein levels (Fig. 1F, G).

### Knockdown of USP40 inhibits HCC cell proliferation, migration and stemness

**Fig. 2:**
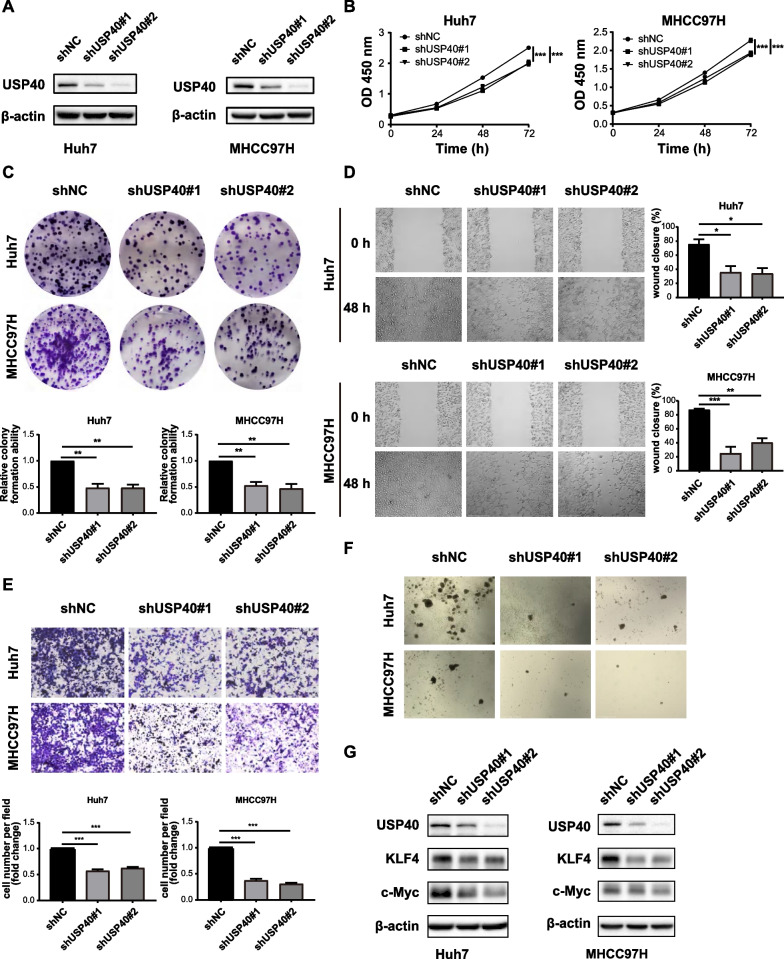
Knockdown of USP40 inhibits HCC cell proliferation, migration and stemness. **A** Huh7 and MHCC97H stably knocked down USP40 cell lines were established by lentivirus infection, and western blotting was performed to detect USP40 protein expression. **B** CCK-8 and **C** colony formation experiments revealed that USP40 depletion decreased the growth of Huh7 and MHCC97H cells. **D** Wound healing and **E** transwell results showed that the migration ability of Huh7 and MHCC97H cells decreased after USP40 depletion. **F** The capacity of HCC cells to form spheroids was reduced after knockdown of USP40. **G** Knockdown of USP40 inhibited the expression of tumor stem cell indicators c-Myc and KLF4. Data were displayed with typical pictures of three independent experiments. *P < 0.05, **P < 0.01, ***P < 0.001

To study the role of USP40 in HCC, stable USP40 knockdown cell lines of Huh7 and MHCC97H were constructed using lentiviral transfection. Western blotting verified that USP40 was effectively silenced in HCC cells (Fig. 2A). CCK-8 and colony formation experiments revealed that USP40 depletion suppressed HCC cell growth (Fig. 2B, C). Wound healing and transwell experiments were used to determine the influence of USP40 on the migration capacity of HCC cells. The results demonstrated that the capacity of HCC cells to migrate was significantly weakened after USP40 knockdown (Fig. 2D, E). To explore the function of USP40 in HCC cell stemness, we detected spheroid forming capacity and stemness-related proteins expression. As depicted in Fig. 2F, the capacity of HCC cells to form spheroids was drastically diminished after USP40 knockdown, with smaller spheroids observed in the USP40 knockdown group. Meanwhile, the levels of stemness-related proteins including c-Myc and KLF4 were drastically reduced in the USP40 depletion group (Fig. 2G). Overall, USP40 knockdown inhibited HCC cell proliferation, migration and stemness.

### Overexpression of USP40 promotes HCC cell proliferation, migration and stemness

**Fig. 3:**
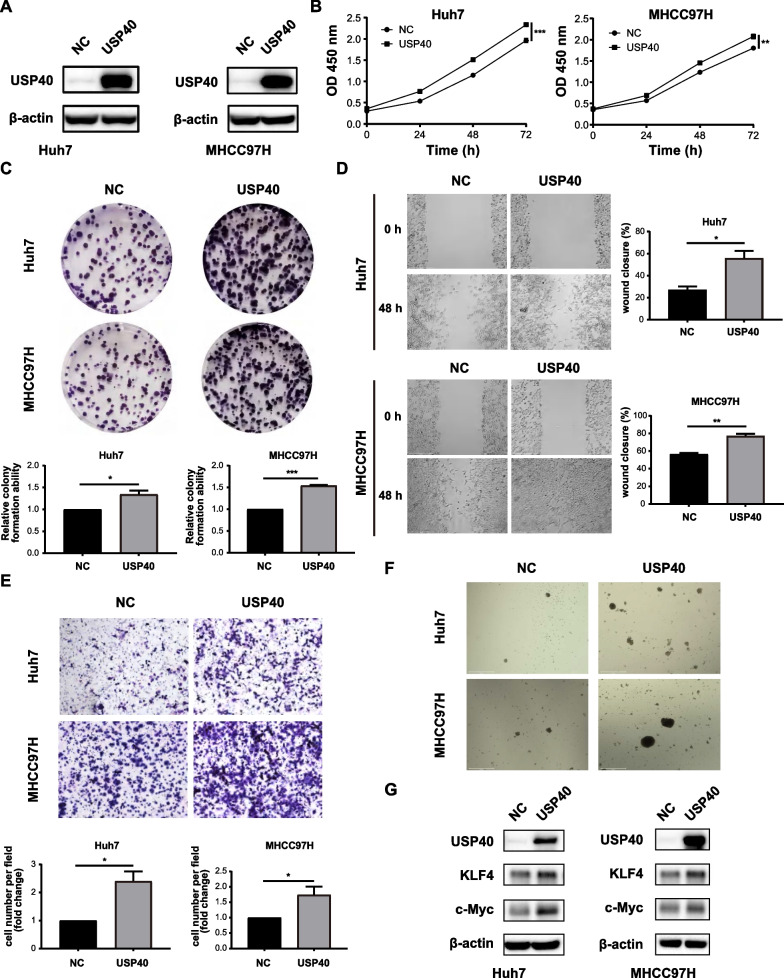
Overexpression of USP40 promotes HCC cell proliferation, migration and stemness. **A** Huh7 and MHCC97H stably overexpressed USP40 cell lines were established by lentivirus infection, and western blotting was performed to detect the USP40 protein expression. **B** CCK-8 and **C** colony formation experiments revealed that USP40 overexpression promoted the growth of Huh7 and MHCC97H cells. **D** Wound healing and **E** transwell results suggested that USP40 overexpression increased the migratory capacity of Huh7 and MHCC97H cells. **F** The capacity of HCC cells to form spheroids was improved after USP40 overexpression. **G** Overexpression of USP40 increased the expression of tumor stem cell indicators c-Myc and KLF4. Data were displayed with typical pictures of three independent experiments. *P < 0.05, **P < 0.01, ***P < 0.001

To further confirm the function of USP40 in HCC, Huh7 and MHCC97H cell lines stably overexpressed USP40 were constructed by lentivirus transfection. Western blotting showed that USP40 was stably overexpressed in HCC cells (Fig. 3A). CCK-8 and colony formation results showed that USP40 upregulation enhanced HCC cell growth (Fig. 3B, C). Furthermore, wound healing and tranwell assays confirmed that USP40 upregulation also promoted HCC cell migration (Fig. 3D, E). Contrary to the results of USP40 knockdown, the spheroid size increased after USP40 overexpression, indicating that USP40 promoted spheroid formation ability (Fig. 3F). Meanwhile, overexpression of USP40 elevated the levels of stemness-related proteins, including c-Myc and KLF4 (Fig. 3G). In general, our findings suggest that USP40 upregulation can enhance HCC cell proliferation, migration and stemness.

### USP40 interacts with Claudin1 and regulates Claudin1 expression

**Fig. 4:**
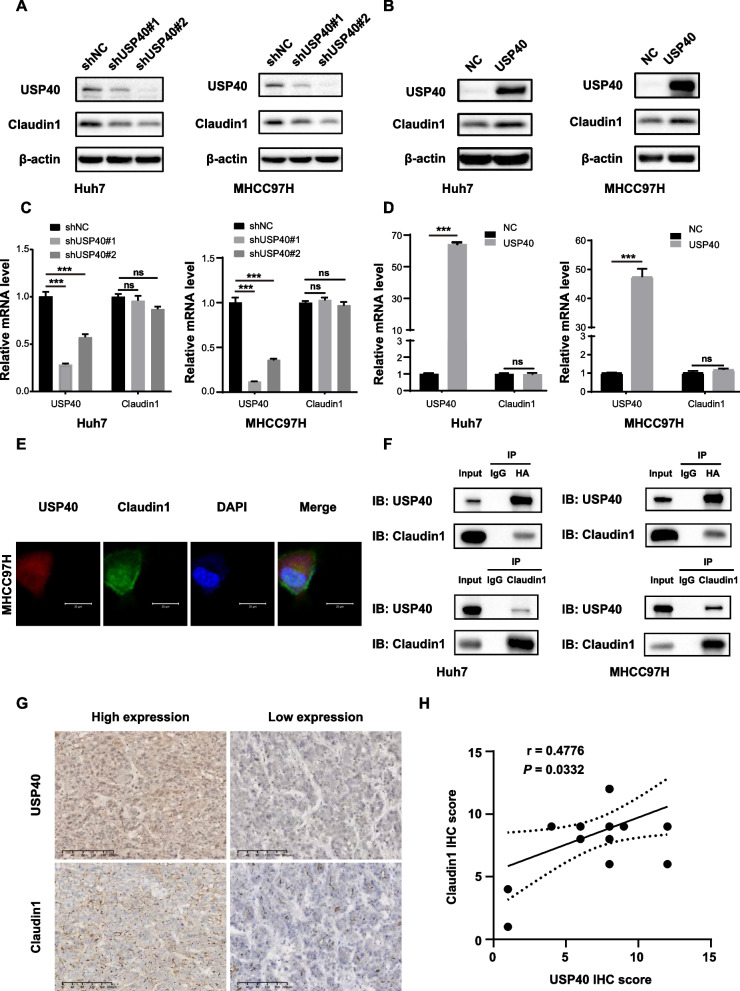
USP40 interacts with Claudin1 and regulates Claudin1 expression. **A**–**B** Western blotting was performed to determine the impact of knockdown or overexpression of USP40 on the expression of Claudin1 protein levels. **C**–**D** RT-qPCR was utilized to assess the influence of knockdown or overexpression of USP40 on Claudin1 mRNA levels. **E** Using USP40 and Claudin1 antibodies, the cellular location of USP40 (red) and Claudin1 (green) in HCC cells was examined by immunofluorescent staining and photographed by laser confocal microscopy. Scale bar, 50 μm. **F** HA-USP40 and Claudin1 plasmids were co-transfected into Huh7 and MHCC97H cells, and Co-IP was employed to determine the interactions among USP40 and Claudin1. **G** Representative IHC staining pictures of USP40 and Claudin1 were displayed. Scale bar, 200 μm. **H** The correlation between USP40 and Claudin1 expression in HCC tissues was determined by Pearson’s correlation (n = 20). ns: not significant. Data were displayed with typical pictures of three independent experiments. ***P < 0.001

Western blotting was performed to detect the impact of USP40 on Claudin1 protein expression. We observed that Claudin1 protein expression was dramatically decreased in Huh7 and MHCC97H cells after USP40 knockdown, while it was significantly elevated in USP40 overexpressed cells (Fig. 4A, B). However, RT-qPCR results suggested that either suppression or overexpression of USP40 had no impact on mRNA level of Claudin1 (Fig. 4C, D), indicating that the regulation of Claudin1 by USP40 was focus on the post-translational level. Immunofluorescence staining further revealed the co-localization of USP40 and Claudin1 in the cytoplasm of MHCC97H cells (Fig. 4E). Then we transfected HA-USP40 and Claudin1 plasmids into HCC cells, Co-IP findings demonstrated the interactions between USP40 and Claudin1 (Fig. 4F), which was consistent with the immunofluorescence staining results. Furthermore, USP40 and Claudin1 protein expression were positively correlated in HCC specimens, according to IHC analysis (Fig. 4G, H).

### USP40 stabilizes Claudin1 by promoting its deubiquitination

Since USP40 was a DUB and regulated Claudin1 expression on protein level but not RNA level, we supposed that USP40 may regulate Claudin1 through its deubiquitination activity.

**Fig. 5:**
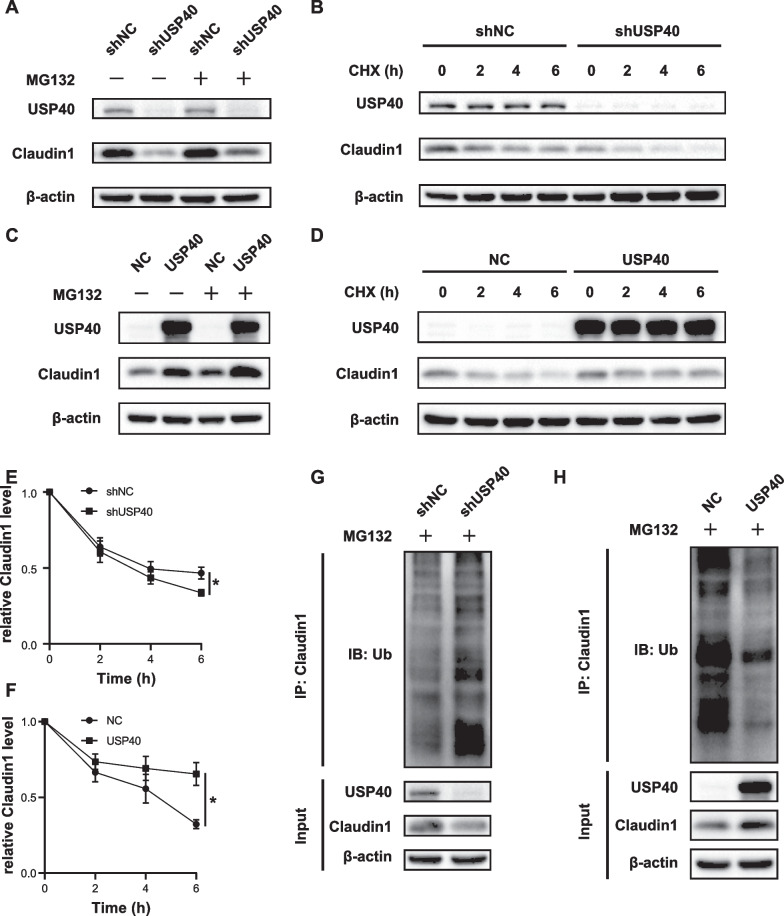
USP40 stabilizes Claudin1 by promoting its de-ubiquitination. **A** Stable USP40 knockdown MHCC97H cells and negative control (NC) cells were treated by MG132 (10 μM) for 8 h, and then Claudin1 expression was tested by western blotting. **B** CHX assay was conducted to detect the half-life of Claudin1 in USP40 knockdown and NC cells. Each group was treated by CHX (100 µg/mL) for a specified time. Next, proteins were collected and then Claudin1 expression was tested by western blotting. **C** MHCC97H cells stably overexpressed USP40 and NC cells were treated by MG132 (10 μM) for 8 h, and then Claudin1 expression was tested by western blotting. **D** CHX assay was conducted to detect the half-life of Claudin1 in USP40 overexpressed and NC cells. **E**–**F** Quantitative evaluation of the half-life of the Claudin1 protein. **G** Effect of USP40 silencing on Claudin1 ubiquitination. Lysates from MHCC97H cells were immunoprecipitated using Claudin1 antibody and Claudin1 polyubiquitination was examined using western blotting. **H** Effect of USP40 overexpression on Claudin1 ubiquitination in MHCC97H cells. Data were displayed with typical pictures of three independent experiments. *P < 0.05

To further investigate the molecular mechanism, we added proteasome specific inhibitor MG132 to HCC cells and found that MG132 protected Claudin1 from degradation (Fig. 5A, C), suggesting that the degradation of Claudin1 protein is carried out through the proteasomal pathway. In addition, to confirm that USP40 can regulate Claudin1 stability, the half-life of Claudin1 protein in USP40 knockdown or USP40 overexpression cells was analyzed by CHX assay. We observed that Claudin1 protein level in the USP40 knockdown group decreased faster compared to the control group, while USP40 overexpression enhanced the protein stability of Claudin1 (Fig. 5B, D, E, F).

Since USP40 is a DUB, we further explored the function of USP40 in Claudin1 polyubiquitination. Ubiquitination assay revealed that USP40 knockdown increased the polyubiquitination of Claudin1, whereas USP40 overexpression reduced the polyubiquitination of Claudin1 (Fig. 5G, H).

In summary, our above findings showed that USP40 maintains the stability of Claudin1 and cleaves the polyubiquitin chain of Claudin1 through its deubiquitination activity, which demonstrated that USP40 regulates Claudin1 protein expression through post-translational modification (PTM).

### USP40 promotes HCC progression by regulating Claudin1

**Fig. 6:**
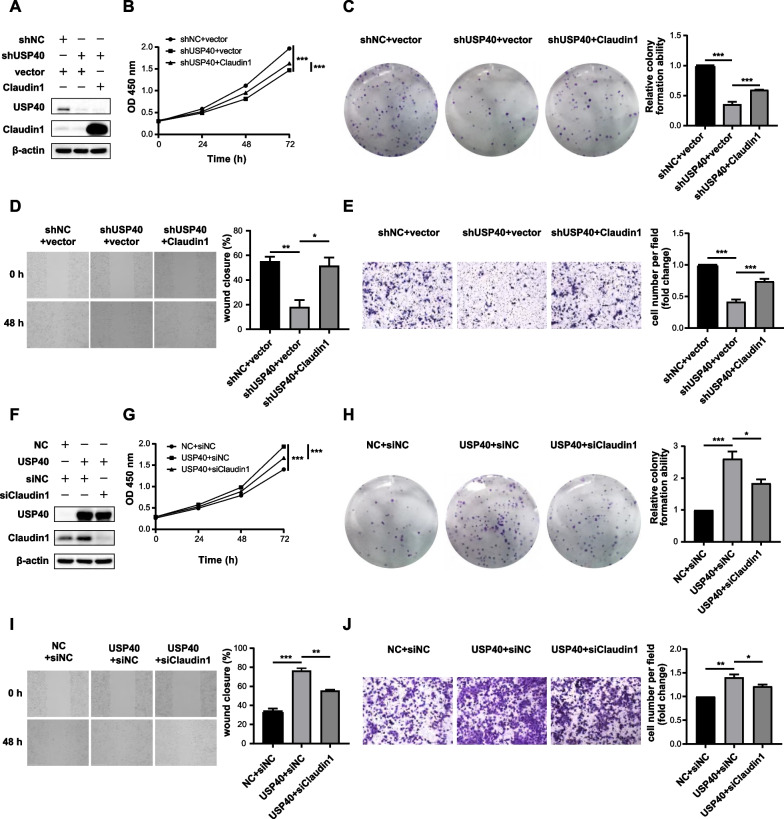
USP40 promotes HCC progression by regulating Claudin1. **A** Knockdown of USP40 decreased Claudin1 protein levels, and this effect was reversed by Claudin1 overexpression. Claudin1 or vector plasmids were transfected into MHCC97H cells with stable USP40 knockdown, and expression of USP40 and Claudin1 was detected by western blotting. The proliferative and migratory capacity of MHCC97H cells was detected by CCK-8 (**B**), colony formation (**C**), wound healing (**D**) and transwell (**E**), respectively. **F** SiClaudin1 or siNC were transfected into MHCC97H cells that USP40 stably overexpressed. USP40 and Claudin1 expression was detected by western blotting. The proliferative and migratory ability of MHCC97H cells was also tested by CCK-8 (**G**), colony formation (**H**), wound healing (**I**) and transwell (**J**), respectively. Data were displayed with typical pictures of three independent experiments. *P < 0.05, **P < 0.01, ***P < 0.001

To investigate whether Claudin1 is participated in USP40-mediated HCC cell growth and migration, several rescue experiments were conducted. MHCC97H cells with stable knockdown of USP40 were transfected with Claudin1 plasmid or vector, and western blotting showed that USP40 silencing reduced Claudin1 protein levels, which could be restored by overexpressing Claudin1 (Fig. 6A). CCK-8 test displayed that USP40 depletion dramatically suppressed HCC cell growth, and Claudin1 overexpression partially reversed this growth (Fig. 6B), which was also verified by colony formation assay (Fig. 6C). Furthermore, wound healing and transwell experiments revealed that USP40 silencing mediated cell migration suppression was partly restored by overexpressing Claudin1 (Fig. 6D, E).

Then, we transfected siClaudin1 or siNC to MHCC97H cells stably overexpressed USP40 (Fig. 6F), CCK-8 and colony formation showed that the impact of USP40 upregulation on promoting cell proliferation could be limited by Claudin1 suppression (Fig. 6G, H). Similarly, wound healing and transwell revealed that USP40 upregulation led to increased migration ability of HCC cells, which could be reversed by knocking down Claudin1 (Fig. 6I, J). Therefore, we inferred that USP40 facilitates HCC cell proliferation and migration by regulating Claudin1.

### USP40 promotes tumor growth in vivo

**Fig. 7:**
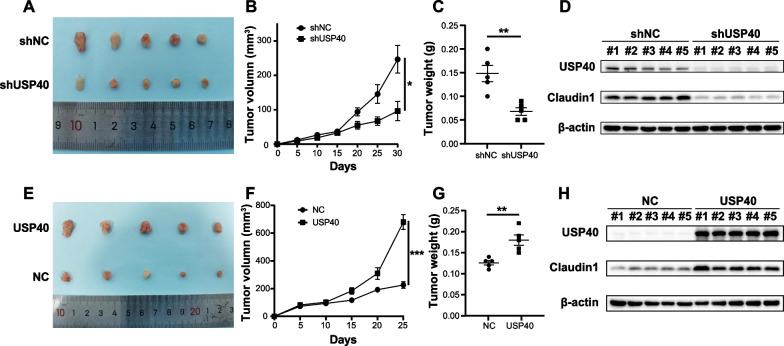
USP40 promotes tumor growth in vivo. **A** Stable transfection of MHCC97H cells with shNC or shUSP40 were subcutaneously inoculated into nude mice, and the mice were euthanized 30 days later and the tumors were dissected to obtain images of tumors. **B**, **C** showed the tumor size and weight of nude mice. **D** USP40 and Claudin1 expression in tumor samples was tested by western blotting. **E** The picture displayed subcutaneous tumors formed by MHCC97H cells in overexpressed USP40 or NC group. **F**, **G** showed the tumor size and weight of nude mice. **H** USP40 and Claudin1 expression in tumor samples was examined by western blotting. Data were displayed with typical pictures of three independent experiments. *P < 0.05, **P < 0.01, ***P < 0.001

To examine the function of USP40 in tumor growth in vivo, MHCC97H cells were used to establish the subcutaneous xenograft nude mice model. During the research, we recorded the length and width of the tumor every 5 days. Finally, western blotting was used to detect USP40 and Claudin1 expression in tumor samples. The findings indicated that the tumor volume and weight were diminished in the USP40 knockdown group compared to the corresponding control group (Fig. 7A–C). On the contrary, tumor size and weight were larger in the USP40 overexpression group compared to the control group (Fig. 7E–G). Western blotting analysis of tumor tissues suggested that USP40 knockdown suppressed Claudin1 expression, and USP40 overexpression promoted Claudin1 expression (Fig. 7D, H), which was in accordance with the findings of in vitro experiments. In summary, we confirmed that USP40 facilitates HCC growth in vivo.

## Discussion

**Fig. 8:**
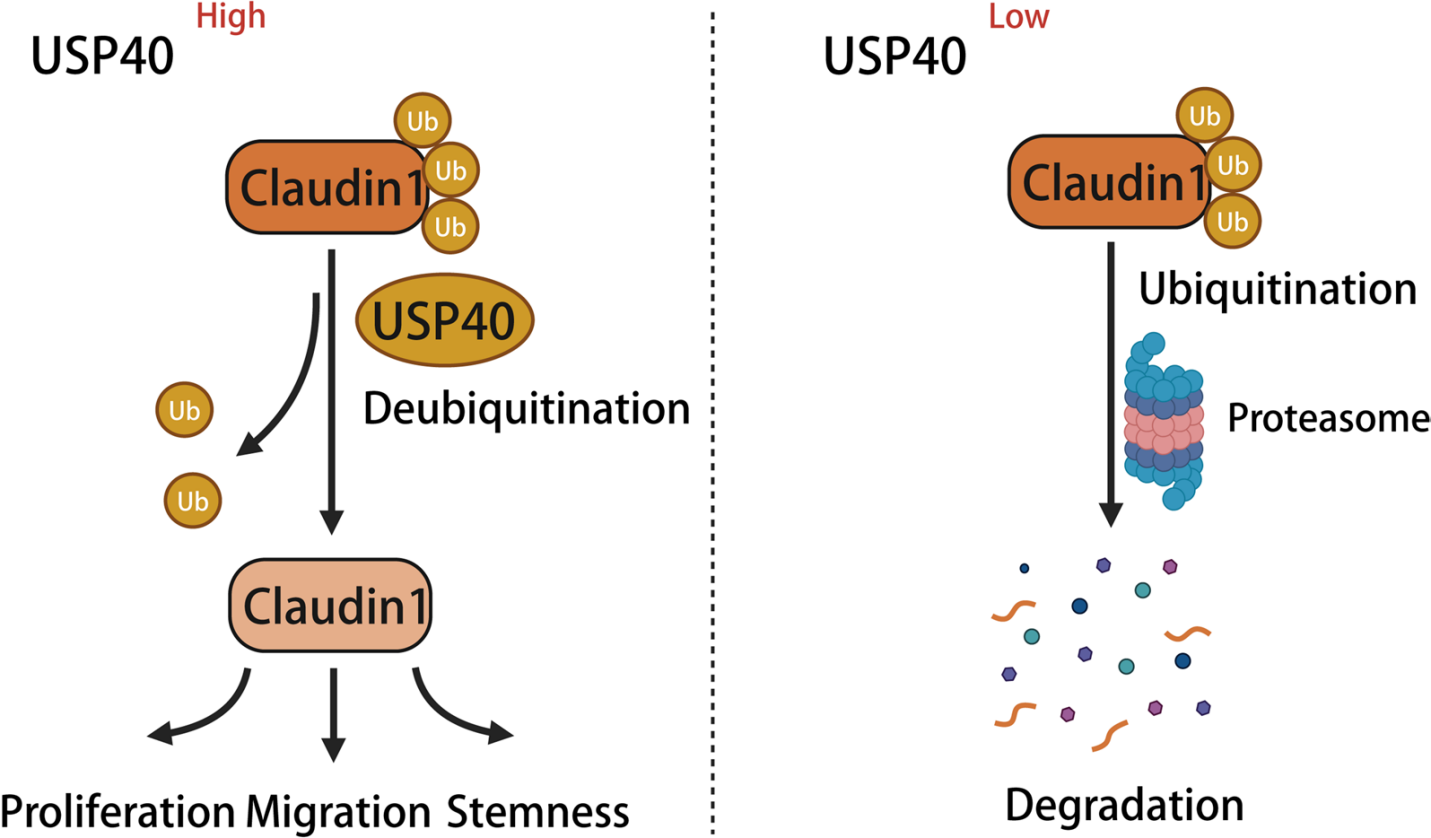
Scheme for the regulatory mechanism of USP40 on Claudin1. This schematic reveals that USP40 HCC promotes proliferation, migration, and stemness by regulating Claudin1 expression

In our research, we identified a novel DUB USP40. We observed that USP40 was elevated in human HCC samples and that patients with elevated USP40 expression showed poor prognosis. In addition, USP40 knockdown inhibited HCC cell growth, migration and stemness. Mechanistic experiments revealed that USP40 interacted with Claudin1 and increased protein stability by inhibiting polyubiquitination of Claudin1 in HCC cells (Fig. 8).

USP40 belongs to the USP family, and its role in malignant tumors has been poorly studied. Through whole exon sequencing, Xu et al. reported a case of lung adenocarcinoma caused by USP40 mutations, indicating that the gene plays a crucial driving role [22]. Studies have shown that USP40 deubiquitinates and stabilizes CFLAR_L_, and GMEB1 interacts with USP40 to maintain CFLAR_L_ and prevent apoptosis in NSCLC [11].

Claudin1 belongs to the claudins family and participates in the construction of tight junctions. Claudin1 is also expressed at non-tight junctions, such as the basolateral membrane of hepatocytes. In malignant tumors, Claudin1 is abnormally localized. Claudin1 has been found to be overexpressed in colon cancer, and there is also abnormal localization from cell membrane to nucleus and cytoplasm [13]. Through immunofluorescence co-localization experiments, we found that Claudin1 was localized in the cytoplasm and even nucleus of MHCC97H cells, which preliminarily confirmed the ectopic expression of Claudin1, and its cytoplasmic localization may promote its carcinogenesis. Kim et al. found that Claudin1 in RBFOX3-positive cells was higher than in RBFOX3-negative cells in lung tissues, and RBFOX3 increased the stability of Claudin1 by weakening its ubiquitination [23]. Similarly, our study confirmed that Claudin1 is degraded by the ubiquitin–proteasome system in HCC, and USP40 inhibits Claudin1 polyubiquitination to increase its stability.

c-Myc is an oncogenic transcription factor participated in biological functions including cancer cell proliferation, EMT, cancer stemness, metastasis, and metabolic reprogramming [24, 25]. The transcription factor KLF4 has been reported to promote cancer cell stemness in various types of cancers [26–29]. Karagonlar et al. discovered that KLF4 induced a CSC-like behavior in non-CSCs by elevating EpCAM and E-CAD expression in Huh7 cells [30]. Researchers found that deletion of Claudin1 in NSCLC inhibited p-AKT expression and diminished cancer cell stemness via inhibiting AKT activation [31]. Similarly, Roehlen et al. confirmed that Claudin1 overexpression is related to stemness in HCC. Moreover, Claudin1 specific antibody effectively inhibited cancer stemness and EMT [32]. We confirmed that silencing USP40 limited the sphere-forming capacity of HCC cells and decreased c-Myc and KLF4 expression. However, whether there is a regulatory relationship between Claudin1 and the expression of c-Myc and KLF4 needs to be further investigated. In addition, the specific ubiquitination site of Claudin1 and its binding region with USP40 are worthy of further study.

Here, we described the role of USP40 in HCC for the first time. USP40 was elevated in HCC and is an unfavorable prognostic biomarker for HCC. USP40 facilitated cell growth, migration and stemness in vitro and in vivo. Further, we firstly identified Claudin1 as a novel target of USP40. USP40 regulates Claudin1 expression through the post-translational pathway. USP40 could interact with Claudin1 and deubiquitinates Claudin1 to stabilize its protein level. Functional rescue experiments revealed that USP40 and Claudin1 formed a functional axis to enhance HCC malignant progression.

## Conclusions

In this study, our findings elucidated that USP40 exerts tumor-promoting effects by regulating Claudin1 and targeting the USP40-Claudin1 axis is a promising strategy for HCC therapy.

### Supplementary Information

**Additional file 1**. Supplementary Tables.

## Data Availability

The datasets used and analyzed during the current study are available from the corresponding author on reasonable request.

## References

[1] Sung H, Ferlay J, Siegel RL, Laversanne M, Soerjomataram I, Jemal A, Bray F (2021). Global cancer statistics 2020: GLOBOCAN estimates of incidence and mortality worldwide for 36 cancers in 185 countries. CA: Cancer J Clin.

[2] Allemani C, Weir HK, Carreira H, Harewood R, Spika D, Wang X-S, Bannon F, Ahn JV, Johnson CJ, Bonaventure A, Marcos-Gragera R, Stiller C, Azevedo e Silva G, Chen W-Q, Ogunbiyi OJ, Rachet B, Soeberg MJ, You H, Matsuda T, Bielska-Lasota M, Storm H, Tucker TC, Coleman MP (2015). Global surveillance of cancer survival 1995–2009: analysis of individual data for 25 676 887 patients from 279 population-based registries in 67 countries (CONCORD-2). The Lancet.

[3] Chen W, Zheng R, Baade PD, Zhang S, Zeng H, Bray F, Jemal A, Yu XQ, He J (2016). Cancer statistics in China, 2015. CA: Cancer J Clin.

[4] Llovet JM, Kelley RK, Villanueva A, Singal AG, Pikarsky E, Roayaie S, Lencioni R, Koike K, Zucman-Rossi J, Finn RS (2021). Hepatocellular carcinoma. Nat Rev Dis Prim.

[5] Li Y, Schrodi S, Rowland C, Tacey K, Catanese J, Grupe A (2006). Genetic evidence for ubiquitin-specific proteases USP24 and USP40 as candidate genes for late-onset Parkinson disease. Hum Mutat.

[6] Wu YR, Chen CM, Chen YC, Chao CY, Ro LS, Fung HC, Hsiao YC, Hu FJ, Lee-Chen GJ (2010). Ubiquitin specific proteases USP24 and USP40 and ubiquitin thiolesterase UCHL1 polymorphisms have synergic effect on the risk of Parkinson’s disease among Taiwanese. Clin Chim Acta: Inte J Clin Chem.

[7] Cleynen I, Vazeille E, Artieda M, Verspaget HW, Szczypiorska M, Bringer MA, Lakatos PL, Seibold F, Parnell K, Weersma RK, Mahachie John JM, Morgan-Walsh R, Staelens D, Arijs I, De Hertogh G, Müller S, Tordai A, Hommes DW, Ahmad T, Wijmenga C, Pender S, Rutgeerts P, Van Steen K, Lottaz D, Vermeire S, Darfeuille-Michaud A (2014). Genetic and microbial factors modulating the ubiquitin proteasome system in inflammatory bowel disease. Gut.

[8] Takagi H, Nishibori Y, Katayama K, Katada T, Takahashi S, Kiuchi Z, Takahashi SI, Kamei H, Kawakami H, Akimoto Y, Kudo A, Asanuma K, Takematsu H, Yan K (2017). USP40 gene knockdown disrupts glomerular permeability in zebrafish. Am J Physiol Renal Physiol.

[9] Takahashi S, Fukuhara D, Kimura T, Fukutomi T, Tanaka E, Mikami N, Hada I, Takematsu H, Nishibori Y, Akimoto Y, Kiyonari H, Abe T, Huber O, Yan K (2022). USP40 deubiquitinates HINT1 and stabilizes p53 in podocyte damage. Biochem Biophys Res Commun.

[10] Hwang J, Kim H, Han J, Lee J, Hong S, Kim S, Yoon SK, Choi K, Yang J, Park U, Kim K, Yim K, Kim Y, Choi Y (2022). Identification of survival-specific genes in clear cell renal cell carcinoma using a customized next-generation sequencing gene panel. J Personal Med.

[11] An W, Yao S, Sun X, Hou Z, Lin Y, Su L, Liu X (2019). Glucocorticoid modulatory element-binding protein 1 (GMEB1) interacts with the de-ubiquitinase USP40 to stabilize CFLAR(L) and inhibit apoptosis in human non-small cell lung cancer cells. J Exp Clin Cancer Res: CR.

[12] Mailly L, Baumert TF (2020). Hepatitis C virus infection and tight junction proteins: the ties that bind. Biochim Biophys Acta (BBA) Biomembr.

[13] Dhawan P (2005). Claudin-1 regulates cellular transformation and metastatic behavior in colon cancer. J Clin Investig.

[14] Tokés AM, Kulka J, Paku S, Szik A, Páska C, Novák PK, Szilák L, Kiss A, Bögi K, Schaff Z (2005). Claudin-1, -3 and -4 proteins and mRNA expression in benign and malignant breast lesions: a research study. Breast Cancer Res: BCR.

[15] Bouchagier KA, Assimakopoulos SF, Karavias DD, Maroulis I, Tzelepi V, Kalofonos H, Karavias DD, Kardamakis D, Scopa CD, Tsamandas AC (2014). Expression of claudins-1, -4, -5, -7 and occludin in hepatocellular carcinoma and their relation with classic clinicopathological features and patients’ survival. In vivo (Athens, Greece).

[16] Holczbauer Á, Gyöngyösi B, Lotz G, Törzsök P, Kaposi-Novák P, Szijártó A, Tátrai P, Kupcsulik P, Schaff Z, Kiss A (2014). Increased expression of Claudin-1 and Claudin-7 in liver cirrhosis and hepatocellular carcinoma. Pathol Oncol Res.

[17] Chang JW, Seo ST, Im MA, Won H-R, Liu L, Oh C, Jin YL, Piao Y, Kim HJ, Kim JT, Jung S-N, Koo BS (2022). Claudin-1 mediates progression by regulating EMT through AMPK/TGF-β signaling in head and neck squamous cell carcinoma. Transl Res.

[18] Suh Y, Yoon CH, Kim RK, Lim EJ, Oh YS, Hwang SG, An S, Yoon G, Gye MC, Yi JM, Kim MJ, Lee SJ (2012). Claudin-1 induces epithelial–mesenchymal transition through activation of the c-Abl-ERK signaling pathway in human liver cells. Oncogene.

[19] Stebbing J, Filipović A, Giamas G (2013). Claudin-1 as a promoter of EMT in hepatocellular carcinoma. Oncogene.

[20] Wang W, Lei Y, Zhang G, Li X, Yuan J, Li T, Zhong W, Zhang Y, Tan X, Song G (2023). USP39 stabilizes β-catenin by deubiquitination and suppressing E3 ligase TRIM26 pre-mRNA maturation to promote HCC progression. Cell Death Dis.

[21] Wang D, Li Z, Li X, Yan C, Yang H, Zhuang T, Wang X, Zang Y, Liu Z, Wang T, Jiang R, Su P, Zhu J, Ding Y (2022). DUB1 suppresses Hippo signaling by modulating TAZ protein expression in gastric cancer. J Exp Clin Cancer Res: CR.

[22] Xu ZH, Wang H, Ji XY, Zhang FX, Gao BL, Hu JA, Zheng J (2019). A detrimental mutation on USP40 unlocks the tumorigenesis in a rare case of lung cancer. Int J Clin Exp Pathol.

[23] Kim Y-E, Choi S, Kim JO, Kim KK (2017). RBFOX3 regulates Claudin-1 expression in human lung tissue via attenuation of proteasomal degradation. Biosci Rep.

[24] Miller M, Thomas SD, Islam A, Muench D, Sedoris K (2012). c-Myc and cancer metabolism. Clin Cancer Res: Off J Am Assoc Cancer Res.

[25] Yoshida GJ (2018). Emerging roles of Myc in stem cell biology and novel tumor therapies. J Exp Clin Cancer Res: CR.

[26] Yu F, Li J, Chen H, Fu J, Ray S, Huang S, Zheng H, Ai W (2011). Kruppel-like factor 4 (KLF4) is required for maintenance of breast cancer stem cells and for cell migration and invasion. Oncogene.

[27] Zhu XY, Wang L, Luan SH, Zhang HS, Huang WT, Wang NH (2014). The PGI-KLF4 pathway regulates self-renewal of glioma stem cells residing in the mesenchymal niches in human gliomas. Neoplasma.

[28] Qi XT, Li YL, Zhang YQ, Xu T, Lu B, Fang L, Gao JQ, Yu LS, Zhu DF, Yang B, He QJ, Ying MD (2019). KLF4 functions as an oncogene in promoting cancer stem cell-like characteristics in osteosarcoma cells. Acta Pharmacol Sin.

[29] Leng Z, Li Y, Zhou G, Lv X, Ai W, Li J, Hou L (2020). Krüppel-like factor 4 regulates stemness and mesenchymal properties of colorectal cancer stem cells through the TGF-β1/Smad/snail pathway. J Cell Mol Med.

[30] Firtina Karagonlar Z, Akbari S, Karabicici M, Sahin E, Tercan Avci S, Ersoy N, Eren Ates K, Balli T, Karacicek B, Kaplan KN, Celiker C, Atabey N, Erdal E (2020). A novel function for KLF4 in modulating the de-differentiation of EpCAM−/CD133− nonstem cells into EpCAM+/CD133+ liver cancer stem cells in HCC cell line HuH7. Cells.

[31] Jia Z, Wang K, Duan Y, Hu K, Zhang Y, Wang M, Xiao K, Liu S, Pan Z, Ding X (2022). Claudin1 decrease induced by 1,25-dihydroxy-vitamin D3 potentiates gefitinib resistance therapy through inhibiting AKT activation-mediated cancer stem-like properties in NSCLC cells. Cell Death Discov.

[32] Roehlen N, Muller M, Nehme Z, Crouchet E, Jühling F, Del Zompo F, Cherradi S, Duong FHT, Almeida N, Saviano A, Fernández-Vaquero M, Riedl T, El Saghire H, Durand SC, Ponsolles C, Oudot MA, Martin R, Brignon N, Felli E, Pessaux P, Lallement A, Davidson I, Bandiera S, Thumann C, Marchand P, Moll S, Nicolay B, Bardeesy N, Hoshida Y, Heikenwälder M, Iacone R, Toso A, Meyer M, Elson G, Schweighoffer T, Teixeira G, Zeisel MB, Laquerriere P, Lupberger J, Schuster C, Mailly L, Baumert TF (2023). Treatment of HCC with claudin-1-specific antibodies suppresses carcinogenic signaling and reprograms the tumor microenvironment. J Hepatol.

