# Supplementary material for: USP40 promotes hepatocellular carcinoma cell proliferation, migration and stemness by deubiquitinating and stabilizing Claudin1
Source: Biol Direct. 2024 Feb 2;19:13. doi: 10.1186/s13062-024-00456-3 (PMC10837946; doi:10.1186/s13062-024-00456-3)
Supplement: Supplementary file 1 — Additional file 1. Supplementary Tables. [file 13062_2024_456_MOESM1_ESM.docx]

**Table S1.** The shRNA sequences used in the study.

| **shRNA** | **sequences (5'-3')** |
| --- | --- |
| shUSP40#1 | TCCCCACTGGTTTGATATAAA |
| shUSP40#2 | TGGGCCCTCAGTATCATTTCT |
| shNC | AGGCCATTACCATGTATATAT |

**Table S2.** The siRNA sequences used for transfection.

| **siRNA** | **sense (5'-3')** | **antisense (5'-3')** |
| --- | --- | --- |
| siClaudin1 | GAAUCGUUCAAGAAUUCUATT | UAGAAUUCUUGAACGAUUCTT |
| siNC | UUCUCCGAACGUGUCACGUTT | ACGUGACACGUUCGGAGAATT |

**Table S3.** Primer sequences used for RT-qPCR in the study.

| **Gene** | **Forward primer (5'-3')** | **Reverse primer (5'-3')** |
| --- | --- | --- |
| USP40 | CAGAAAGCGTGTGGGATTTGACC | GTGAAGTCCTGCTGGTACAAGC |
| Claudin1 | GTCTTTGACTCCTTGCTGAATCTG | CACCTCATCGTCTTCCAAGCAC |
| β-actin | CACCATTGGCAATGAGCGGTTC | AGGTCTTTGCGGATGTCCACGT |
